# Investigating the ability of non-invasive measures of cardiac output to detect a reduction in blood volume resulting from venesection in spontaneously breathing subjects

**DOI:** 10.1186/s13049-018-0571-5

**Published:** 2018-12-04

**Authors:** Benjamin Mothibe Bussmann, William Hulme, Andrew Tang, Tim Harris

**Affiliations:** 1 Emergency Department, Royal London Hospital, Barts Health NHS Trust, London, UK; 2 Faculty of Biology, Medicine and Health, University of Manchester, Manchester, UK; 3 Department of anaesthesia at Norfolk and Norwich University Hospital, Colney Lane, Norwich, UK; 4 Queen Mary University of London, London, UK

**Keywords:** Cardiac output, Stroke volume, Inferior vena cava collapsibility index, USCOM, LiDCO, CNAP, Venesection, Flow time, Carotid blood flow, Circulating blood volume

## Abstract

**Background:**

Monitoring cardiac output (CO) in shocked patients provides key etiological information and can be used to guide fluid resuscitation to improve patient outcomes. Previously this relied on invasive monitoring, restricting its use in the Emergency Department (ED) setting. The development of non-invasive devices (such as LiDCOrapid^v2^ with CNAP™ and USCOM 1A), and ultrasound based measurements (Transthoracic echocardiography, inferior vena cava collapsibility index (IVCCI), carotid artery blood flow (CABF) and carotid artery corrected flow time (FTc)) enables stroke volume (SV) and CO to be measured non-invasively in the ED. We investigated the ability of these techniques to detect a change in CO resulting from a 500 ml reduction in circulating blood volume (CBV) following venesection in spontaneously breathing subjects. Additionally, we investigated if using incentive spirometry to standardise inspiratory effort improved the accuracy of IVC based measurements in spontaneously breathing subjects.

**Methods:**

We recorded blood pressure, heart rate, IVCCI, CABF, FTc, transthoracic echocardiographic (TTE) SV and CO, USCOM 1A SV and CO, LIDCOrapidv2 SV, CO, Stroke volume variation (SVV) and pulse pressure variation (PPV) in 40 subjects immediately before and after venesection. The Log-Odds and coefficient of variation of the difference between pre- and post-venesection values for each technique were used to compare their ability to consistently detect CO changes resulting from a reduction in CBV resulting from venesection.

**Results:**

TTE consistently detected a reduction in CO associated with venesection with an average decrease in measured CO of 0.86 L/min (95% CI 0.61 to 1.12) across subjects. None of the other investigated techniques changed in a consistent manner following venesection. The use of incentive spirometry improved the consistency with which IVC ultrasound was able to detect a reduction in CBV.

**Conclusions:**

In a population of spontaneously breathing patients, TTE is able to consistency detect a reduction in CO associated with venesection.

## Background

Knowledge of haemodynamic status is key for successful fluid resuscitation of the shocked patient [1]. Clinicians commonly use changes in heart rate and blood pressure to guide fluid resuscitation, but these may remain normal in the early stages of shock and are not reliable predictors of adequate fluid resuscitation [2]. However, knowledge of changes in CO / SV in response to fluid administration may assist in determining the volume of resuscitation fluid delivered, improving patient outcomes [1].

Previously monitoring of CO / SV required costly, invasive devices restricting their use to an intensive care setting. More recently ultrasound is increasingly being used in the ED and offers a range of non-invasive tools to estimate CO [3]. Common techniques include IVCCI[4], CABF [5], FTc [6] and TTE spectral Doppler to calculate the stroke volume [7].

Currently TTE is the recommend modality for the initial evaluation of shocked patients due to its ability to accurately estimate and track changes in CO [8] and assist in identifying the aetiology of the dominant shock syndrome [9]. Additionally recent work has shown that TTE is reliable even when performed by clinicians with limited sonographic training [10].

IVCCI measurements have been shown to predict fluid responsiveness in mechanically ventilated patients [4], and predict hypovolaemia in trauma patients [11]. However, in spontaneously breathing patients, they have been shown to be unreliable [12], probably due to effects variation in inspiratory effort and intra-pleural pressures on IVC diameters [4].

The carotid artery measurements CABF and FTc both are novel techniques with only limited published research to date concerning their ability to predict fluid responsiveness in shocked patients [5, 6, 13].

Recently several dedicated non-invasive cardiac output monitors have been developed, including LIDCOrapid^v2^ with CNAP™ (continuous non-invasive arterial pressure) [14] and USCOM 1A (Ultrasound Cardiac Output Monitor) [15]. CNAP™ uses an inflatable pressure cuff placed on the fingers to measure arterial pressure in the digital arteries, using a volume clamp method. The resulting arterial pressure waveform is analysed though pulse contour analysis to provide beat to beat CO and SV values. USCOM 1A estimates CO and SV using continuous wave Doppler to determine blood flow in the ascending aorta.

LiDCOrapid^v2^ with CNAP™ has only been validated on invasively ventilated patients in intensive care or intra-operative settings [16] and we are not aware of any studies exploring its performance in spontaneously breathing patients.

USCOM 1A has been studied for use in spontaneously breathing patients where it has shown good inter-rater reliability and ease to learn [17, 18]. However, validation studies have produced mixed results [19].

The primary aim of this study is to compare the ability of TTE, USCOM 1A, LiDCOrapid^v2^ with CNAP™, IVCCI and carotid artery blood flow measures to detect the change in CO resulting from 500 ml of blood loss in spontaneously breathing subjects undergoing venesection. Secondly we tested the hypothesis that the use of incentive spirometer to standardise inspiratory effort would improve the accuracy of IVC measurements in spontaneously breathing subjects.

## Methods

### Study design and setting

This is an observational study using patients with a diagnosis of haemochomatosis or polycythemia attending for routine venesection, based in the haematology outpatient department in a regional teaching hospital. Over a period of 12 months a convenience sample of 42 patients attending venesection were included in this study. Exclusion criteria were BMI greater than 40, atrial fibrillation, ventricular dysfunction, known severe or symptomatic valvular heart disease, pregnancy, recent abdominal surgery and inability to lie flat.

### Protocol

We recorded participants height, weight, gender and age on the day of enrolment. Venesection of 500 ml of blood was performed by haematology nursing staff as per local protocol. Immediately before and after venesection the following measurements were taken: upper arm oscillometric blood pressure, heart rate, IVC diameters, carotid artery blood flow, FTc, TTE SV and CO, USCOM 1A SV and CO, LIDCOrapid^v2^ with CNAP™ SV, CO, SVV and PPV. Subjects remained in a supine position for the duration of the study.

All readings were taken by a single operator with Royal College of Emergency Medicine (RCEM) Level 1 (Core) ultrasound certification. The investigator was trained by device manufacturers in their use. Additionally, a log of 50 practice scans in each ultrasound study technique was completed prior to initiation of this study. Competency in each technique was confirmed through triggered assessment by the regional RCEM lead for ultrasound training. All ultrasound measurements were made with the Sonosite Edge® ultrasound system (Sonosite Inc., Bothell Washington, USA).

### Measurements

IVC measurements were taken using a 5–1 MHz phased array transducer (Sonosite Inc., Bothell Washington, USA), in the long axis 3–5 cm from the right atrium. Minimum (IVCi) and maximum (IVCe) anterior-posterior diameters during quiet respiration were determined through frame-by-frame analysis of saved B mode cine-loop recordings using Phillips DICOM viewer 3.0 (Philips Medical Systems, Eindhoven, Netherlands). IVCCI was calculated as IVCe – IVCi/ IVCe.

We also measured IVC diameters during a controlled inspiratory manoeuvre. Subjects were instructed to inhale for as long as possible through a DHD CliniFLO® Flow Breathing Exerciser whilst keeping the flow ball within its target zone, producing a controlled and sustained inspiratory effort of 400 ml/s. The time taken from initiation of controlled inspiration to complete IVC collapse was measured (Tcol).

Carotid artery blood flow measurements were taken using a 13-6 MHz linear array transducer (Sonosite Inc., Bothell Washington, USA). The right carotid artery was identified through its pulsation, Doppler flow and lack of compressibility. Carotid artery maximum and minimum intimal-to-intimal anterior-posterior diameters were measured in long axis during systole, 2 cm proximal to the carotid bifurcation.

Doppler measurements were made from the centre of the vessel with a 3 mm gate with a maximum angle of isonation of 60 degrees.

Flow time measurements were made from saved spectral Doppler waveform images using Phillips DICOM viewer 3.0. Corrected flow time (FTc) was defined as flow time/√(r-r interval) [6].

TTE was performed with a 5–1 MHz phased array transducer (Sonosite Inc., Bothell Washington, USA). Maximum left ventricular outflow tract (LVOT) diameters during systole were determine in the parasternal long axis view. Measurements were taken at the site of attachment of the aortic valve leaflets using frame-by-frame analysis of saved B mode cine-loop recordings on Phillips DICOM viewer 3.0. Aortic valve area was calculated as πr^2^ where r = LVOT diameter/2. Pulsed wave Doppler measurements of the velocity time integral (VTI) were recorded with a 3 mm gate placed in the left LVOT in an apical 5-chamber view. SV was calculated as: (aortic valve area x VTI). CO was calculated as (SV x HR).

USCOM 1A (USCOM Ltd., Australia) CO and SV measurements were taken via a suprasternal notch approach. Only traces with a minimum Fremantle score of 4/6 were used [17]. CO and SV were calculated by integral software using patient demographic information provided.

LIDCOrapid^v2^ with CNAP™ (CNSystems Medizintechnik AG, Graz, Austria) was set up as recommended by the manufacturer. LiDCOrapid^v2^ was calibrated with an upper arm oscillometric blood pressure value obtained immediately prior to setting up the device and again after completion of venesection. LiDCOview^pro^ software was used to extract SVV, PPV, SV and CO values.

### Statistical analysis

We compared techniques according the consistency with which they were able to detect a change in CO resulting from venesection, based on the difference between their pre- and post-venesection values, *x*_*∆*_ = *x*_*post*_ − *x*_*pre*_. We did not determine sensitivity as this would require a predefined magnitude at which a change in a reading can be considered significant. However, to our knowledge no such predefined magnitude exists for tracking reductions in CO, so any values used would be arbitrary and of unknown clinical significance.

First, we determined the consistency of direction of change following venesection. The number of cases where *x*_*∆*_ increases is compared with the number of cases where *x*_*∆*_ decreases by using a modification of the logarithm of the odds ratio (LO) which accounts for cases where *x*_*∆*_ = 0. This is a robust measure of the number of increases versus number of decreases in *x* after venesection which can be directly compared across variables. Larger values indicate greater consistency of direction of change.

Secondly, we determined the consistency of magnitude of change resulting from venesection. The average of *x*_*∆*_ across patients is divided by the standard deviation of *x*_*∆*_. This is simply the inverse of the coefficient of variation (1/CV) of *x*_*∆*_. Larger values indicate greater consistency of magnitude of change.

Formally, the two measures are defined as follows:

$$ \frac{1}{CV}=\frac{\mathrm{mean}\left({x}_{\Delta }\right)}{\mathrm{std}.\mathrm{dev}\left({x}_{\Delta }\right)} $$

and

$$ LO=\log \left[\frac{{\left[{x}_{\Delta }\right]}_{+}+\frac{{\left[{x}_{\Delta }\right]}_0}{2}\ }{{\left[{x}_{\Delta }\right]}_{-}+\frac{{\left[{x}_{\Delta }\right]}_0}{2}}\right] $$

where *x*_*∆*_ = *x*_*post*_ − *x*_*pre*_, [*x*_*∆*_]_+_ =  ∑ (*x*_*∆*_ > 0), [*x*_*∆*_]_−_ =  ∑ (*x*_*∆*_ < 0), [*x*_*∆*_]_0_ =  ∑ (*x*_*∆*_ = 0).

Both measures of consistency can range from −∞ to ∞, with values further away from zero indicating better performance.

Patients where *x*_*pre*_ or *x*_*post*_ were missing were excluded on a variable-by-variable basis.

Confidence intervals were obtained via bootstrapping (10,000 bootstrap replicates, taking the 0.025 and 0.975 quantiles).

The statistical software R (version 3.5.1) was used for all statistical analyses and outputs (R Core Team (2018). R: A language and environment for statistical computing. R Foundation for Statistical Computing, Vienna, Austria).

## Results

42 consecutive patients gave consent and were enrolled in the study. Two were excluded because they did not complete venesection.

Participant characteristics are summarised in Table 1. All post-venesection measurements where completed within an average time of 16 min (range 11–34) after completion of venesection.

**Table 1:** Summary of participant characteristics

Summary of participant Characteristics	
Total	40
Male	37
Female	3
Age (years)	50.8 (range 26–72)
Height (cm)	175.5 (range 157–193)
Weight (kg)	86.2 (range 52–130)
BMI (kg/m^2^)	27.9 (range 18.4–39.7)

**Fig. 1:**
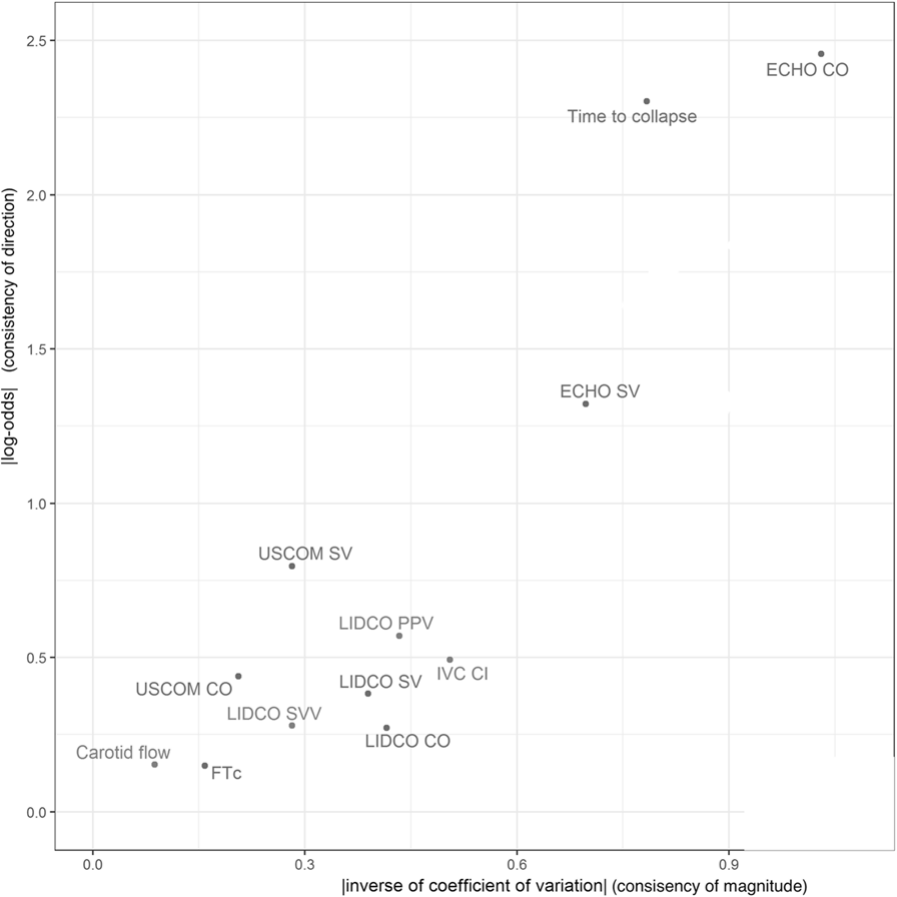
This figure shows a plot of the absolute value of the inverse of the coefficient of variation plotted against the absolute value of the log-odds ratio. Variables in the upper right area of this plot showed the greatest consistancy in change resulting from venesection. CO Cardiac output, SV stroke volume, LIDCO LiDCOrapid^v2^ with CNAP™, SVV stroke volume variation, PPV pulse pressure variation, FTc corrected carotid artery flow time, IVCCI inferior vena cava collapsibility index, ECHO transthoracic echocardiography

A summary of all results is shown in Table 2. Due to difficulty in image acquisition or technical device errors it was not possible to obtain paired data sets for all 40 included participants with every technique investigated. The number of interpretable paired data sets (*n*) included in the final analysis for each technique are shown in Table 2. For a visual comparison of the overall performance of each technique according to our two measures of consistency (1/CV and LO) we produced a plot of 1/CV versus LO (Fig. 1).

**Table 2:** Table of results for all variables measured pre- and post-venesection

Measurement (units)	n	# increased following venesection	# decreased following venesection	# no change following venesection	pre-venesection mean	post-venesection mean	Average Change (95% CI)	Inverse Coefficient of Variation (1/CV) (95% CI)	Log-odds (95% CI)
Heart Rate (bpm)	40	7	31	2	77.30	71.47	−5.83 (−7.72, −3.88)	− 0.92 (− 1.37, − 0.59)	− 1.39 (− 2.35, − 0.73)
Diastolic blood pressure (mmHg)	40	11	27	2	74.83	71.30	−3.52 (− 5.85, −1.15)	− 0.46 (− 0.90, − 0.14)	− 0.85 (− 1.64, − 0.25)
Systolic blood pressure (mmHg)	40	6	32	2	136.25	125.38	− 10.88 (− 14.57, − 7.35)	− 0.93 (− 1.28, − 0.67)	− 1.55 (− 2.51, − 0.85)
Mean Arterial Pressure (mmHg)	40	5	34	1	97.78	91.33	−6.45 (−8.57, −4.22)	− 0.91 (− 1.50, − 0.52)	− 1.84 (− 2.94, − 1.10)
IVCe (cm)	29	4	25	0	1.76	1.49	− 0.26 (− 0.37, − 0.16)	− 0.87 (− 1.26, − 0.57)	−1.83 (− 3.33, − 0.97)
IVCi (cm)	29	2	22	5	0.94	0.69	− 0.25 (− 0.37, − 0.14)	−0.80 (− 1.18, − 0.57)	−1.70 (− 2.91, − 0.97)
IVCCI %	29	16	9	4	48	59	10 (3.9, 19)	0.51 (0.30, 0.73)	0.49 (−0.21, 1.24)
Tcol (seconds)	22	2	20	0	2.02	1.267	−0.75 (−1.15, − 0.39)	−0.78 (− 1.23, − 0.47)	−2.30 (-Inf, − 1.22)
Carotid artery blood flow (ml/min)	39	21	18	0	720.1	731.8	11.4 (−29.6, 52.1)	0.09 (−0.22, 0.44)	0.15 (−0.47, 0.81)
r-r interval (seconds)	40	12	18	10	0.90	0.89	−0.0092 (− 0.027, 0.0090)	−0.16 (− 0.49, 0.15)	−0.30 (− 0.85, 0.25)
Flow time (seconds)	40	12	19	9	0.31	0.31	−0.0048 (− 0.011, 0.0018)	−0.22 (− 0.54, 0.09)	−0.35 (− 0.97, 0.20)
Corrected flow time (seconds)	40	18	21	1	0.33	0.33	−0.0040 (− 0.012, 0.0037)	−0.16 (− 0.48, 0.15)	−0.15 (− 0.79, 0.46)
ECHO VTI (cm)	38	8	30	0	21.87	20.34	−1.47 (−2.11, −0.85)	−0.73 (− 1.09, − 0.45)	−1.32 (− 2.46, − 0.65)
ECHO CO (L/min)	38	3	35	0	6.28	5.39	− 0.86 (−1.12, − 0.61)	− 1.03 (− 1.53, − 0.74)	−2.46 (-Inf, − 1.49)
ECHO SV (ml)	38	8	30	0	80.00	74.02	−5.24 (− 7.65, −2.88)	−0.70 (− 1.06, − 0.40)	− 1.32 (− 2.46, − 0.65)
USCOM 1A CO (L/min)	37	14	22	1	4.35	4.13	− 0.18 (− 0.45, 0.10)	−0.21 (− 0.62, 0.11)	−0.44 (− 1.14, 0.22)
USCOM 1A SV (ml)	37	11	25	1	64.87	60.66	−3.19 (− 6.57, 0.57)	−0.28 (− 0.74, 0.05)	−0.80 (− 1.55, − 0.16)
LiDCOrapid^v2^ with CNAP™ CO (L/min)	37	16	21	0	7.29	6.49	−0.80 (− 1.41, − 0.22)	−0.42 (− 0.72, − 0.13)	−0.27 (− 0.99, 0.38)
LiDCOrapid^v2^ with CNAP™ PPV %	36	22	12	2	13.70	16.47	3.00 (0.86, 5.28)	0.43 (0.14, 0.74)	0.57 (−0.06, 1.34)
LiDCOrapid^v2^ with CNAP™ SV (ml)	37	14	21	2	101.22	90.62	−10.59 (− 19.54, − 2.16)	−0.39 (− 0.70, − 0.09)	−0.38 (− 1.06, 0.22)
LiDCOrapid^v2^ with CNAP™ SVV %	36	18	13	5	15.95	17.89	2.08 (−0.17, 4.56)	0.28 (−0.03, 0.57)	0.28 (− 0.34, 0.96)

n is the number of paired scans included in final analysis. #increase and #decrease show the number of measurement that increased and decreased in magnitude following venesection respectively. IVCe maximum inferior vena cava diameter on expiration, IVCi minimum inferior vena cave diameter on inspiration, IVCCI inferior vena cava collapsibility index, Tcol time taken for complete IVC collapse during controlled inspiratory manoeuvre, ECHO transthoracic echocardiography, *VTI* velocity time integral, *CO* cardiac output, *SV* stroke volume, *USCOM* Ultrasound Cardiac Output Monitor, *SVV* stroke volume variation, *PPV* Pulse pressure variation

## Discussion

The aim of this study was to compare the ability of TTE, USCOM 1A, LiDCOrapid^v2^ with CNAP™, IVCCI, CABF and FTc to consistently detect a change in CO resulting from venesection.

Subjects responded to venesection with a reduction in mean arterial pressure (− 6.5 mmHg (95% CI -8.6 to − 4.2)) and HR (− 5.8 bpm (95% CI -7.7 to − 3.9)), similar in magnitude to changes seen in other venesection studues [15, 20].

Of all measures tested, only TTE was able to consistently detect a change in CO and SV following venesection (Fig. 1.). TTE determined CO and SV decreased by 0.86 L/min (95% CI 0.61 to 1.12) and 5.24 ml (95% CI 7.65 to 2.88) respectively, which is similar in magnitude to changes seen in other venesection studies [21]. None of the other measures we tested changed in a consistent manner following venesection (Fig. 1).

USCOM 1A determined changes in CO and SV resulting from venesection were inconsistent in both direction and magnitude of change (Fig. 1.). To our knowledge only one other study used USCOM 1A to measure SV in spontaneously breathing subjects undergoing venesection. O’loughlin et al. demonstrated a significant reduction in SV following venesection of 7.5% of CBV [15]. We were unable to reproduce a significant change in SV despite a similar sized reduction in CBV. This might be explained by differing ages of subjects investigated. Subjects investigated by O’loughlin et al. were much younger than ours (mean age 36.5 years vs 50.8 years in our study) and previous work suggests that USCOM 1A is less accurate in older patients, likely due unfolding and calcification of the aorta with age [22].

To our knowledge we are the first to investigate the non-invasive CNAP™ technology on spontaneously breathing subjects. We found that all variables recorded with LiDCOrapid^V2^ with CNAP™ (CO, SV, PPV, SVV) changed in an inconsistent manner following venesection (Fig. 1.). We did not formally control subjects’ finger positions so it is possible that finger movements affected the readings taken by the CNAP™ finger cuffs. However, we feel this would also be the case in a population of ED patients.

Carotid artery measurements performed poorly (Fig. 1) with an approximately equal proportion of increases and decreases in CABF and FTc following venesection (Table 2), suggesting that any observed changes were unrelated to CBV.

We were unable to reproduce findings by Mackenzie et al. showing an average decrease in FTc of 21 milliseconds following blood donation [13]. However, this change is very small and was observed over a larger sample size than ours (70 subjects), so it is possible that our study was underpowered to detect such small changes. Additionally subjects investigated by Mackenzie et al. were much younger (median age of 29 years) than our subjects, so age related changes such as atherosclerosis might also account for differing results.

The only study to-date investigating CABF suggested it can be used to predict fluid responsiveness when combined with a passive leg raise manoeuvre [5]. This however was performed on haemodynamically unstable patients requiring vasopressor support and mechanical ventilation. Our results suggest more work is required to define any potential role for FTc or carotid blood flow in guiding fluid therapy.

IVCCI measurements increased by 10% (95% CI 3.9 to 19%) following venesection which is similar in magnitude to changes seen in other venesection studies [21, 23, 24]. Despite this overall average increase, the observed changes in IVCCI were inconsistent across subjects, varying greatly in magnitude and direction of change. This inconsistency is reflected in the literature with conflicting results [20, 21] and poor inter-observer reliability reported for this technique [10], suggesting that IVCCI must be used with caution in spontaneously breathing subjects [4].

Additionally, as IVC diameters reach the point of collapse with blood loss, the IVCi approaches a zero-value causing IVCCI to plateau at 100%. Beyond this point IVCCI remains constant at 100% despite further decreases in CBV and can thus not be used to track further reductions in CBV. Of note, in this study 7 subjects had complete IVC collapse following venesection on only 500 ml of blood, suggesting that even a small reduction in CBV can lead to compete IVC collapse and render this marker insensitive to further blood loss.

It is suggested that spontaneous breathing results in variable tidal volumes, and intrathoracic pressures, leading to inconsistent changes in IVCCI [4, 12]. We used an incentive spirometer to standardise the tidal volumes of spontaneously breathing subjects and measured the time taken for complete IVC collapse to occur during a controlled and sustained inspiratory effort (Tcol). This novel measure of IVC dynamics consistently decreasing by 0.75 s (95% CI 0.39 to 1.15) following venesection. This supports the theory that variable tidal volumes during inspiration contribute to the inconsistencies seen in IVCCI measurements in spontaneously breathing subjects.

### Limitations

This was a pilot study carried out by a single un-blinded investigator with no intra-observer reliability analysis carried prior to the study. There was no control group and sample size was small. Results will need to be confirmed in a larger blinded study with multiple operators.

Our study population was largely male possibly due to the increased prevalence of haemochromatosis in this population so our results may not be generalisable.

The effect of haemochromatosis on cardiac function was not formally assessed at the time of enrolment, so it is possible that some subjects may have had altered cardiac function due to cardiac disease. However, all subjects were asymptomatic and we excluded any subjects with known prior cardiac diagnoses.

We assumed subjects were euvolaemic prior to venesection and we did not control fluid intake prior to venesection.

We did not assess the correlation across the different measurement techniques used in this study nor compare their absolute values to an accepted reference standard. This is because the primary aim of our study was to assess ability of each method to track a change in CO, rather than compare absolute values.

## Conclusion

In our population of spontaneously breathing subjects, TTE was able to consistently detect changes in CO occurring in response to blood loss. USCOM 1A and LiDCOrapid^V2^ with CNAP™ based measures were inconsistent with inappropriately varying magnitudes. Similarly changes in IVCCI were too inconsistent for clinical use. However, combining IVC ultrasound with incentive spirometry produced a consistent measure of IVC diameters. This novel technique requires further assessment but may prove a clinically useful method of tracking fluid status.

## Abbreviations

CABF: Carotid artery blood flow
CBV: Circulating blood volume
CNAP™: Continuous non-invasive arterial pressure finger cuff
CO: Cardiac output
CV: Coefficient of variation of the mean
FTc: Carotid artery corrected flow time
HR: Heart rate
IVCCI: Inferior vena cava collapsibility index
IVCe: Maximum inferior vena cava diameter on expiration
IVCi: Minimum inferior vena cava diameter on inspiration
LIDCOrapid^v2^: Cardiac output monitor by CNSystems Medizintechnik AG, Graz, Austria
LO: Log Odds
PPV: Pulse pressure variation
SV: Stroke volume
SVV: Stroke volume variation
TTE: Transthoracic echocardiography
USCOM 1A: Ultrasound cardiac output monitor by USCOM Ltd., Australia
